# Navigating uncertain waters: 12 tips for medical department social media engagement under shifting platform landscapes

**DOI:** 10.12688/mep.20175.1

**Published:** 2024-03-20

**Authors:** Zachary Van Roy, Kelly A. Cawcutt, Jasmine R. Marcelin

**Affiliations:** 1 College of Medicine, University of Nebraska Medical Center, Omaha, Nebraska, 68198, USA; 2 Division of Infectious Diseases, University of Nebraska Medical Center, Omaha, Nebraska, 68198, USA

**Keywords:** social media, engagement, tips, department strategy

## Abstract

Social media has revolutionized how society receives and transmits information in the digital age, including healthcare. While the future of social media platforms remains uncertain, the benefits of social media for healthcare organizations, departments, and divisions are clear when compared to traditional communication methods, including improved recruitment efforts, increased promotion of faculty work, rapid dissemination of information and education innovations, and accelerated professional networking. Consequently, preferred platforms may shift but the benefits of social media likely cement it, in one form or another, in medical education and society at large. The strategic development of a social media presence at the department level can be opaque and overwhelming amidst varying mediums, yet the benefits of use have never been more important. Here, we present 12 tips for developing a successful social media presence as a healthcare department, addressing the creation of a purposeful social media strategy and crucial considerations for any platform, current or future, at all levels of development.

## Introduction

In the three decades since the World Wide Web launched in the public domain, it has thoroughly restructured how society shares information. The academic medicine migration to social media (SoMe) is evident in the advent of “medical Twitter” (#MedTwitter) ^1^ and various other loosely defined subsets of the SoMe sphere dedicated to medical topics and specialties. Most medical institutions and many departments have an active Facebook page ^2^, and an increasing number are expanding into other SoMe platforms, including X (formerly Twitter), Instagram, YouTube ^3^, and LinkedIn ^4^. SoMe interventions have been linked to increased knowledge ^5, 6^, empathy ^7^, reflective writing skills ^8, 9^, and engagement with educators within the medical community ^10^. The healthcare workforce additionally experiences unrestricted emersion in medical knowledge and advancements, historically restricted to publications or conference attendance ^11, 12^. Further, research articles shared on SoMe had significantly higher readership than non-promoted work by a factor of 5–10 fold ^13^, with additional increases in article altmetric scores and subsequent citations ^14^. SoMe is also a valuable advocacy tool for healthcare professionals employing mission-based engagement ^15^, and additionally functions as a means to build one’s medical professional network ^16^, especially for women and underrepresented populations ^12, 17^.

Despite these benefits, there are also potential harms to engaging on social media. Studies conducted before and during the COVID-19 pandemic demonstrated increased experiences of targeted harassment and discrimination among women users and those from demographic backgrounds underrepresented in medicine ^18, 19^. This has led many healthcare professionals to consider leaving SoMe despite the potential professional benefits ^18, 20^. Concomitantly, concerns regarding the spread of health misinformation on SoMe platforms have recently been systematically verified ^21^, showing the highest levels of misinformation on X (formerly Twitter) commonly surrounding tobacco and drug use, vaccines, and non-communicable diseases. While individual engagement in these platforms is ubiquitous among healthcare professionals in academic medical centers, there has been a slow uptake in recognition of the value of meaningful professional engagement for promotion and tenure. However, blueprints for accurately documenting these activities on curriculum vitae ^22^ and incorporating these contributions into the promotion and tenure process have been published ^23^.

Further complicating effective SoMe use is the turbulent stability of social media websites when viewed through a longitudinal lens. Unlike departmental blogs hosted locally on institutional servers or newsletter communications distributed via email, SoMe engagement relies on external services which may be destabilized due to any combination of social, political, or financial factors. Illustrating this, the SoMe platform X (formerly Twitter) has undergone significant changes with new ownership, including newfound algorithmic amplification of anger-associated posts ^24, 25^ and anti-science movements ^26^, leading many to question the long-term viability of the site ^20^. With this and the implementation of a mandatory paid tier for X site utilization in some countries which could be universal in the future ^27^, many programs without a current SoMe strategy may feel discouraged from installing a SoMe presence or those with a current account may question whether it is worth maintaining their activity on a platform with an uncertain future. While these are valid concerns, they do not diminish the clear benefits of SoMe engagement on the department level. As new platforms are formed, they are likely to have similar core features and functionality as current offerings ^28^. Therefore, with small adjustments, a thoughtfully-devised SoMe strategy can be fluid across platforms, enabling departments to shift with the SoMe landscape without losing engagement.

Recognizing the potential benefits and harms of SoMe engagement, there has never been a better time to develop an active, thoughtful, and generalized SoMe strategy designed to work across platforms at the department level to support, amplify, and, to some extent, protect faculty. We present 12 tips for establishing a successful social media presence as a department, recognizing the steep learning curve presented by new and ever-shifting SoMe platforms. The first six tips act as a stepwise operational guide to setting up a SoMe presence informed by the literature and using the University of Nebraska Medical Center Infectious Disease Division (UNMC ID) SoMe experience as a case study. The last six tips represent essential troubleshooting considerations for any department accounts, especially with an uncertain and fluid SoMe landscape.

## Tip 1

### Setting goals – Defining the ‘why’

The benefits of implementing SoMe at the department level are too broad for a one-size-fits-all approach to establishing an impactful online presence. The first step for developing a department’s online presence is similar to building an individual presence – answering the question, “Why is this account needed”? Identify strategic goals for the use of social media. One goal, as identified for UNMC ID, may be to increase the national recognition and readership of department faculty members’ work. Another may be engaging in more productive faculty or fellowship recruitment. Identify specific shortcomings you wish to improve upon as a department and pen them in ink. This will help inform your SoMe strategy as you develop your platform. The realized benefits will depend on how the platforms are used to achieve the goals.

## Tip 2

### Assemble a team

With these goals in mind, the next step is establishing a SoMe team responsible for executing the strategy. Team members should have strengths that apply to digital media and the preset department goals, and each member should have distinct responsibilities. For example, if the strategy involves implementing multiple platforms, primary responsibility for each platform may be delegated to a different team member or cross posting may be more easily facilitated via an automated 3 ^rd^ party service. Your team may need to grow with the advent of new platforms and members may need to shift to alternative platforms should one become nonviable. Importantly, early and sustained adoption of new services in parallel with established platforms are most likely to yield account success. Content curation takes time and effort, so FTE-protected time, monetary compensation, and administrative support should be budgeted wherever possible. Identify and amplify team accomplishments in real-time and make room for team creative license. When competing for the readers’ attention, encouraging creative use of SoMe will foster the development of attention-grabbing and share-worthy content necessary for platform success.

## Tip 3

### Plan a route

After assembling the team, designing a SoMe strategy involves defining five different variables: audience(s), platform(s), SoMe-specific goal(s), message(s), and how success will be defined.

The audience may be as narrow as subspecialists within the department or as broad as any member of the public interested in healthcare (or, more likely, both). Identification of the target population will help you craft appropriate and relevant content. SoMe efforts at UNMC ID focused on both infectious disease (ID) specialists and the public with a mix of nuanced infectious disease content and broadly applicable ID posts.

The choice of platform(s) will be heavily influenced by the target audience(s) and team skill sets/resources. Currently, patients are generally more likely to frequent Facebook or YouTube pages ^29^. At the same time, academic healthcare professionals from other institutions have previously been described as primarily using X (formerly Twitter) or alternative social networks (see #MedTwitter ^1^ or #IDTwitter). A blog is best used for targeting local healthcare professionals and the lay public familiar with the institution. Close attention should be paid to shifting SoMe trends and the strategy should be adjusted accordingly. Occasionally, a predicted mass exodus from a SoMe platform may not materialize, so quick adoption and slow abandonment of accounts is likely to be a fruitful component of SoMe strategy.

SoMe objectives should be targeted at achieving strategic goals by providing a mix of content that the target audience(s) can search for, understand, and follow. In practice, this usually means posting a combination of expert-targeted and public-targeted content. The message should be unique, with content that stimulates conversation without sacrificing informational integrity. Consistent messaging will project the department’s image as an expert resource for content, clinical information, and potential speakers in your field. The UNMCID blog ^30^ was initially designed to showcase faculty introductions, achievements, and publications. Over time, the modified strategy included article summaries, journal club recaps, pharmacy reviews, special interest pieces, and pieces on current ID trends to better capture the attention of a larger audience.

Success can be measured through SoMe metrics and progress toward the department goals. SoMe metric goals may include a posts-per-month quota, follower/subscriber count, or article access counts. These metrics measure the effectiveness of SoMe implementation and execution. Department goal tracking should be individualized but could include tracking faculty speaker invitations, number of applications to trainee or faculty positions, or collaboration invitations over time.

## Tip 4

### All-aboard: Work to get faculty and public relations (PR) department to buy into SoMe to maximize impact

The first and most important partner in initially setting up a department’s SoMe presence is the institution’s public relations (PR) or strategic communications department. They will likely be most concerned with ensuring the team has a formalized strategy and can help teams understand their organization’s SoMe policies. They may ask to provide initial oversight over a calendar of prescheduled posts to ensure the new SoMe account meets the institution’s standards and can be a significant asset as they can advise how to best communicate with the chosen audience.

Department faculty (and staff, trainees, depending on the department) will likely be the strongest ally in amplifying content to reach a wider audience. Recruit many interested members to join SoMe and foster engagement with formal Faculty Development programs to provide broader SoMe training for faculty, staff, and trainees.

## Tip 5

### Get posting!

With a thoughtful SoMe plan and a capable and supported team, trust the plan and personnel to execute your strategy. Pay close attention to feedback; note what works well and what doesn’t. A quick list of strategic elements that boost audience engagement includes frequent and relevant posts, original content, regular interaction with others’ content, use of media and links in posts, providing reliable information, responding to audience questions, and tagging others in posts when appropriate ^31, 32^.

## Tip 6

### Course correct when necessary

Carefully monitor metrics during the first weeks and months to determine whether the strategy is working, and which components may need alteration. If readership or engagement is suboptimal, work to expand content distribution through faculty networks or additional SoMe platforms and troubleshoot the approach with PR. If engagement is flourishing, but there is little progress towards department goals, the strategy may not target the correct audience, or content may need to shift. This may take several iterations and experimentation with content. Even with flawless strategy, progress towards many larger department goals through SoMe channels takes time, and teams may have to wait longer to see that return on investment.

## Tip 7

### What to post: a mission-based approach

Balancing creativity with authenticity is always a challenge, but can be achieved with a thoughtful, mission-based approach. This approach involves relating all content to the original mission/goal of the department SoMe account. Engaging with and amplifying professionals and other departments with similar strategic goals and objectives will enhance reach and credibility. Lists can be used to facilitate intentional interaction ^31^. When in doubt, use the platform to amplify achievements from the department’s faculty, staff, and trainees; your audience has chosen to receive your content and is thus interested in department stories. Using hashtags in posts may help to increase content visibility and reach ^33^. Lastly, avoid all arguments on a department account. These are rarely effective and can harm the department’s image ^15^.

## Tip 8

### Always remember Inclusion, Diversity, Access, and Equity (IDA&E)

For all the benefits offered by SoMe, one of the most prominent downsides is the ease at which it allows the propagation of biases, discriminatory attitudes, and misinformation. Never amplify problematic posts. Take great care to be mindful of unconscious biases when creating posts, as even accidental missteps have the potential to be magnified on SoMe. A diverse SoMe team and multiple eyes on posts can help ensure that only responsible and respectful content is shared, and that images and content accurately reflect the diversity of the department, institution, and community served.

## Tip 9

### Common pitfalls

Be aware of and avoid the common pitfalls of SoMe. Institutional accounts are not immune to autonomous ‘bot’ and otherwise disingenuous accounts. Do not engage ^34, 35^.

As a trusted authority in healthcare, content shared by academic medical departments, as trusted healthcare authorities, is likely assumed true by the audience. This includes reposts and shared content from other sources through departmental accounts. Verify that anything posted is true before publishing ^34^, and avoid posting individual opinions from an institutional account ^32^. In addition to harming the department account’s perceived trustworthiness and professionalism, these posts are unlikely to help achieve the prescribed department goals.

Be vigilant and proactive about harassment and keep the institution’s PR department informed about potential safety concerns.

Finally, take extensive care to avoid violating HIPAA. This can sometimes be difficult to navigate in an online world. Luckily, many resources exist in the literature to guide the development of educational posts in a HIPAA-compliant manner ^36^.

## Tip 10

### It takes time

All aspects of a successful SoMe strategy require a significant investment of time and resources to implement successfully. This is likely a more significant time commitment than any single full-time faculty member has to devote. Setting clear and reasonable responsibilities for SoMe team members is essential. Additionally, using efficiency techniques can help, such as scheduling posts ahead of time when possible, to account for schedule fluctuations in your schedule or blocking off dedicated calendar time for curating and creating posts.

Just as it takes time to implement a successful strategy, it also takes time to realize the benefits of SoMe use as a department. Trust that if you build it (SoMe presence) well, they (your audience) will come.

## Tip 11

### Revisit goals often

Even after establishing and implementing an effective strategy, revisiting goals and progress often ensures ongoing success. The SoMe landscape shifts over time, and departments may need to adjust audience, platform, or content to meet these changes. The decision to remain on or leave a platform is fundamentally individual. However, provided you are not diluting your attention to the detriment of content quality or not compromising department or team values, a policy of slow migration to alternative platforms will likely preserve the most readership. SoMe readership trends which shift rapidly away from one platform can equally rapidly shift back. Additionally, your department goals may also change over time, so periodically tweaking the strategy is a healthy component of a strategic plan. At these points, also reassess needs and resources and identify where there are opportunities to request additional support.

## Tip 12

### Cross-post when possible to extend content reach

While there are undeniable benefits to a SoMe presence on multiple platforms, this does not mean you need to also multiply your content creation speed. A focus on quality over quantity of content has proven a lucrative tradeoff for our division. For example, we have found success in sharing blog posts that may be of interest to a broader audience on X (formerly Twitter) concurrently. However, this may not work in the opposite direction; not all SoMe posts translate to useful blog content. Understanding your audience on each platform and cross-posting content to relevant sites whenever possible can help extend the reach of your message and create a cohesive department image that stands alone and is not tied to any particular service. Your audience will not fault you for seeing the same content in two different places. This becomes especially crucial as platforms shift. Maintaining shared content across multiple sites ensures that a single fading platform does not threaten your entire presence.

## Conclusions

In the years since the medical community has had a significant presence on SoMe, the benefits of incorporating its use at the individual, department, and institutional levels are profound. Here, we have presented 12 tips for successfully implementing SoMe at the department level. The first six tips could serve as a roadmap for installing a new departmental SoMe presence, while the last six incorporate best practices for department SoMe accounts at all stages of maturity.

It is important to remember that the cornerstone of a SoMe strategy is your department and your content, not where it is posted. Platforms serve only to mediate content delivery between you and your readers. Therefore, principles that are effective on one platform will likely translate to others. Diversification of platform usage is the best defense against the shifting site allegiances of your audience. However, the addition of alternative platforms in your strategy is more difficult to manage, but it also extends your department’s reach, as new platforms are an opportunity to reach a new audience, benefiting your presence as a whole. In short, a successful strategy involves the thoughtful and intentional definition of your SoMe strategic purposes and goals, the creation and encouragement of a dedicated team with protections for time, talent, and creative license, the recruitment of colleagues to provide and promote content, attentive and careful post creation with consideration for common SoMe pitfalls and IDA&E, and the establishment of multiple SoMe accounts with shared content to form a cohesive department image.

But does this strategy work? Anecdotally, it did for UNMC ID. In the first five years of the division’s SoMe strategy, UNMC ID achieved the goal of increased national presence and recognition. The division saw an increase in national speakers and national society committee members among faculty (particularly in early-career faculty), several new faculty and fellow candidates recruited through SoMe, and the publication of multiple SoMe scholarly works in the literature ^15, 29, 32^. The benefits of SoMe use are expansive, but the learning curve can be steep. We hope these tips will help spark similar success in other departments’ SoMe efforts.

## Data Availability

No data are associated with this article.
