# Supplementary material for: Navigating uncertain waters: 12 tips for medical department social media engagement under shifting platform landscapes
Source: MedEdPublish (2016). 2024 Dec 10;14:12. Originally published 2024 Mar 20. [Version 3] doi: 10.12688/mep.20175.3 (PMC12372415; doi:10.12688/mep.20175.3)
Supplement: Peer review history

# Reviewer report: Reviewer response for version 3

**DOI:** 10.21956/mep.22308.r40538

**Published:** 2025-01-06

**Authors:** Thomas Cochrane

**Affiliations:** 1 The University of Melbourne, Melbourne, Victoria, Australia

This is an interesting article but has significant omissions currently that could be included to increase impact and critical use awareness.

 Start with some SoMe usage stats (rather than simply broad statements of usage) to highlight the reach of these platforms in different countries - also a comparison of the different SoMe platforms in different countries are important to highlight relevant platforms to target as these vary across countries.

**Tip1 - **Explore the growth of LinkedIn and BlueSky as alternatives to X - as BlueSky is now tracked via Altmetrics and has become the default new home of many academics leaving X.

 A short discussion of understanding who owns the key SoMe platforms and their different IP policies is also important - e.g. Facebook owns everything you post on it and Instagram as well; WhatsApp has security issues etc.!

**Tip3 - **Explore the link between SoMe and Altmetrics more deeply as a key part of the SoMe strategy/tips as platforms tracked by various Altmetrics (Altmetrics, Dimensions, PlumX, etc…) are the key for amplifying impact via linking article DOIs that represent peer-reviewed sources of research and information. - this should part of Tip3.

Our sources - Altmetric

- Deneen *et al. *(2022 ^1^)
- Lamba *et al. *(2020 ^2^)
- Huang *et al.* (2018 ^3^)
- Cochrane *et al.* (2018 ^4^)

The link between SoMe platforms, usage stats and Altmetrics are keys to SoMe impact beyond celebrities/influencers.

**Tip5** **-** should include integrate source DOIs (when available - and choose publishing platforms that support assigning DOIs - e.g. Figshare, OJS, OSF, that can then be embedded in Blog posts) into SoMe posts for linking to trusted sources and Altmetrics tracking!

**Tip9 -** include moderation of SoMe posts

If evidence from practice is presented, are all the underlying source data available to ensure full reproducibility?

Not applicable

Is the topic of the practical tips discussed accurately in the context of the current literature

Partly

Are all factual statements correct and adequately supported by citations?

Partly

Are the conclusions drawn balanced and justified on the basis of the presented arguments?

Partly

Are arguments sufficiently supported by evidence from the published literature and/or the authors’ practice?

Partly

Reviewer Expertise:

Social media, educational technology, technology enhanced learning, open educational practice

I confirm that I have read this submission and believe that I have an appropriate level of expertise to confirm that it is of an acceptable scientific standard, however I have significant reservations, as outlined above.

## References

[1] : A Framework for Amplifying the Teaching-Research Nexus Impact: Leveraging Altmetrics via Figshare. *ASCILITE Publications* .2022; 10.14742/apubs.2022.239 10.14742/apubs.2022.239

[2] : Research productivity of health care policy faculty: a cohort study of Harvard Medical School. *Scientometrics* .2020;124(1) : 10.1007/s11192-020-03433-5 107-130 10.1007/s11192-020-03433-5

[3] : A correlation comparison between Altmetric Attention Scores and citations for six PLOS journals. *PLoS One* .2018;13(4) : 10.1371/journal.pone.0194962 e0194962 10.1371/journal.pone.0194962 29621253 PMC5886419

[4] : Technology Enhanced Learning, Research Impact and Open Scholarship. *Australasian Journal of Educational Technology* .2018;34(3) : 10.14742/ajet.4640 10.14742/ajet.4640

# Reviewer report: Reviewer response for version 3

**DOI:** 10.21956/mep.22308.r40492

**Published:** 2025-01-03

**Authors:** Matthew S. Katz

**Affiliations:** 1 Radiation Oncology Associates PA, Lowell, Massachusetts, USA

The edits made by the authors have addressed my prior comments. I have no further questions and think the manuscript reads well and would be a valuable addition to the literature.

If evidence from practice is presented, are all the underlying source data available to ensure full reproducibility?

Not applicable

Is the topic of the practical tips discussed accurately in the context of the current literature

Partly

Are all factual statements correct and adequately supported by citations?

Yes

Are the conclusions drawn balanced and justified on the basis of the presented arguments?

Yes

Are arguments sufficiently supported by evidence from the published literature and/or the authors’ practice?

Partly

Reviewer Expertise:

NA

I confirm that I have read this submission and believe that I have an appropriate level of expertise to confirm that it is of an acceptable scientific standard.

# Reviewer report: Reviewer response for version 3

**DOI:** 10.21956/mep.22308.r40545

**Published:** 2024-12-17

**Authors:** Philomina Pomaah Ofori

**Affiliations:** 1 Ghana Communication Technology University, Accra, Greater Accra Region, Ghana

1. The authors have written a very important masterpiece on how to approach social media platforms as a healthcare department.
2. Although the introduction solely focused on X, there are other platforms for sharing healthcare information. It is vital to discuss other digital platforms and how they enhance information dissemination.
3. Before the abbreviation, write FTE and HIPAA in full.
4. The authors failed to account for the diversity within various healthcare departments. It is important to address that.
5. “Understanding and knowing your audience's interests” could have been a tip, where you will address issues about how the audience perceives SoMe information.

If evidence from practice is presented, are all the underlying source data available to ensure full reproducibility?

Not applicable

Is the topic of the practical tips discussed accurately in the context of the current literature

Yes

Are all factual statements correct and adequately supported by citations?

Yes

Are the conclusions drawn balanced and justified on the basis of the presented arguments?

Yes

Are arguments sufficiently supported by evidence from the published literature and/or the authors’ practice?

Yes

Reviewer Expertise:

My research interest is in digital healthcare and emerging healthcare technologies. My peer-reviewed articles have focused on social media healthcare and digital health information.

I confirm that I have read this submission and believe that I have an appropriate level of expertise to confirm that it is of an acceptable scientific standard.

# Reviewer report: Reviewer response for version 2

**DOI:** 10.21956/mep.22044.r39610

**Published:** 2024-11-30

**Authors:** Matthew S. Katz

**Affiliations:** 1 Radiation Oncology Associates PA, Lowell, Massachusetts, USA

Overall, the authors provide useful guidance on getting started. The main critique is that there should be some team education about ethical use that is compliant with best practices. A specific area of concern would be the risk of sharing stories that undermine patient trust by sharing too much information. This breach of trust can occur even if not disclosing PHI. 

 That cautionary component of protecting patient privacy should still be central to any purpose-based use of social media by healthcare institutions or professionals.

If evidence from practice is presented, are all the underlying source data available to ensure full reproducibility?

Not applicable

Is the topic of the practical tips discussed accurately in the context of the current literature

Partly

Are all factual statements correct and adequately supported by citations?

Yes

Are the conclusions drawn balanced and justified on the basis of the presented arguments?

Yes

Are arguments sufficiently supported by evidence from the published literature and/or the authors’ practice?

Partly

Reviewer Expertise:

social media use in healthcare

I confirm that I have read this submission and believe that I have an appropriate level of expertise to state that I do not consider it to be of an acceptable scientific standard, for reasons outlined above.

# Reviewer report: Reviewer response for version 2

**DOI:** 10.21956/mep.22044.r38666

**Published:** 2024-09-11

**Authors:** Dink Jardine

**Affiliations:** 1 Graduate Medical Education, Charleston Area Medical Center, Charleston, West Virginia, USA

I appreciate the update provided by the group and enjoyed re-reviewing. Thank you.

If evidence from practice is presented, are all the underlying source data available to ensure full reproducibility?

Not applicable

Is the topic of the practical tips discussed accurately in the context of the current literature

Yes

Are all factual statements correct and adequately supported by citations?

Yes

Are the conclusions drawn balanced and justified on the basis of the presented arguments?

Yes

Are arguments sufficiently supported by evidence from the published literature and/or the authors’ practice?

Yes

Reviewer Expertise:

Oto-HNS, Med education, policy and accrediation

I confirm that I have read this submission and believe that I have an appropriate level of expertise to confirm that it is of an acceptable scientific standard.

# Reviewer report: Reviewer response for version 1

**DOI:** 10.21956/mep.21600.r36487

**Published:** 2024-05-31

**Authors:** Dink Jardine

**Affiliations:** 1 Graduate Medical Education, Charleston Area Medical Center, Charleston, West Virginia, USA

I appreciate the practical application of the work and the supported evidence for recommendations.  Admittedly there are few rigorous evidenced base practices available for social media use to support professional activities within medicine and medical education. 

 Some specific considerations include the following:

  Tip 3 - expansion on "how to define success" would be helpful for the reader.  Are their literature references that support any specific metrics or could be used for benchmarking?  Also other platforms success as Reddit/TikTok/Tumblr/Quora/Threads were not included and may be a consideration if a larger update is being considered.  Current reporting in lay press is that Threads has more users than X now. 

 Tip 8-9 may benefit from discussion on how to address if unconscious bias or unintended perception occurs with a specific post.  Are their resources the authors are aware of to turn to if a post becomes a misstep?  Also as expansion of HIPAA discussion to include the ethics of any potentially identifiable information even when not truly HIPAA. 

If evidence from practice is presented, are all the underlying source data available to ensure full reproducibility?

Not applicable

Is the topic of the practical tips discussed accurately in the context of the current literature

Yes

Are all factual statements correct and adequately supported by citations?

Yes

Are the conclusions drawn balanced and justified on the basis of the presented arguments?

Yes

Are arguments sufficiently supported by evidence from the published literature and/or the authors’ practice?

Yes

Reviewer Expertise:

Oto-HNS, Med education, policy and accrediation

I confirm that I have read this submission and believe that I have an appropriate level of expertise to confirm that it is of an acceptable scientific standard, however I have significant reservations, as outlined above.

# Response

**Published:** 2024-08-08

**Authors:** Jasmine Marcelin

**Affiliations:** Internal Medicine, University of Nebraska Medical Center, Omaha, Nebraska, USA

**Thank you for taking the time to review our article and the opportunity to respond. Please find our responses below in point-by-point fashion.**

**Reviewer comment 1: **Tip 3 - expansion on "how to define success" would be helpful for the reader.  Are their literature references that support any specific metrics or could be used for benchmarking?  Also other platforms success as Reddit/TikTok/Tumblr/Quora/Threads were not included and may be a consideration if a larger update is being considered.  Current reporting in lay press is that Threads has more users than X now. 

**Response:** We thank the reviewer for these suggestions, which greatly expanded the impact of Tip 3. We have expanded this section to include the platforms mentioned by the reviewer, as well as to comment on platform features such as ‘verified account status.’ Unfortunately, we could not find any references in the literature objectively assessing the use of certain metrics over others for academic/medical account success benchmarking. We have included a sentence in this paragraph discussing this and bolstering the fact that account success is a highly individualized concept and likely looks different from department to department, which each forms different goals and strategies.

**Reviewer comment 2:** Tip 8-9 may benefit from discussion on how to address if unconscious bias or unintended perception occurs with a specific post.  Are their resources the authors are aware of to turn to if a post becomes a misstep?  Also as expansion of HIPAA discussion to include the ethics of any potentially identifiable information even when not truly HIPAA. 

**Response:** We agree with the reviewer regarding the importance of expanding on detecting unconscious bias in Tip 8. We have added additional commentary discussing implicit bias training and a formalized code of conduct as tools to promote the detection of bias in content. We have also included a statement on recovering from missteps after they occur in Tip 8. Similarly, we have expanded our HIPAA content to include reviewer suggestions regarding advice in sharing information which is not a HIPAA violation but remains ethically dubious

# Reviewer report: Reviewer response for version 1

**DOI:** 10.21956/mep.21600.r36322

**Published:** 2024-04-12

**Authors:** Laurel Fick

**Affiliations:** 1 Internal Medicine Residency Program, Ascension St Vincent Hospital - Indianapolis, Indianapolis, Indiana, USA

This article briefly reviews the role of SoMe in departmental engagement with the public or through professional connections.  While the tips aren't necessarily novel, the pivot to using them to develop departmental accounts (versus residency or individual accounts) hasn't been discussed previously, per my knowledge. The submission article references some of the elements of shifting landscapes within SoMe.

 Some thoughts:

 - Re: **Tip 3** shifting landscape would consider including TikTok as an example of an emerging platform

 - Similarly, curious about the authors' perspective on "verified" status, subscription or only sending content to followers versus public dissemination, or other premium features

 - Consider using "hashtags" on **tip 5** (Bhayani RK; et al., 2020 [Ref 1])

 - Re: **tips 2 and 8**- consider a recommendation for implicit bias training for all members of the team, consider a 'code of conduct' for members to sign 

 - Re: **tip 7**-- "Lists can be used to facilitate intentional interaction"- it isn't clear to me what this means-- what type of lists??

 - Re: online safety for **Tip 9 **would consider commenting about dealing with bullies and monitoring the accounts for spam.  For this tip might also consider the importance of a disclaimer- "this isn't medical advice" or something similar.

 For the conclusions you reference an increased national presence and recognition attributed to the SoMe strategy- do you have the data / graphs to support this?

If evidence from practice is presented, are all the underlying source data available to ensure full reproducibility?

Not applicable

Is the topic of the practical tips discussed accurately in the context of the current literature

Yes

Are all factual statements correct and adequately supported by citations?

Yes

Are the conclusions drawn balanced and justified on the basis of the presented arguments?

Yes

Are arguments sufficiently supported by evidence from the published literature and/or the authors’ practice?

Partly

Reviewer Expertise:

Graduate Medical Education; Physician Wellbeing.

I confirm that I have read this submission and believe that I have an appropriate level of expertise to confirm that it is of an acceptable scientific standard, however I have significant reservations, as outlined above.

## References

[1] : Twelve Tips for Utilizing Residency Program Social Media Accounts for Modified Residency Recruitment. *MedEdPublish (2016)* .2020;9: 10.15694/mep.2020.000178.1 178 10.15694/mep.2020.000178.1 38545456 PMC10967688

# Response

**Published:** 2024-08-08

**Authors:** Jasmine Marcelin

**Affiliations:** Internal Medicine, University of Nebraska Medical Center, Omaha, Nebraska, USA

**Thank you for taking the time to review our article and the opportunity to respond. Please find our responses below in point-by-point fashion.**

**Reviewer comment 1: **Re: **Tip 3** shifting landscape would consider including TikTok as an example of an emerging platform

**Response:** We thank the reviewer for this important suggestion and agree that TikTok’s emergence and recent utilization by health professionals warrant discussion in our manuscript. We have included additional commentary on the rise of TikTok as an emerging platform and included additional references to support this in the revised manuscript.

**Reviewer comment 2:** Similarly, curious about the authors' perspective on "verified" status, subscription or only sending content to followers versus public dissemination, or other premium features

**Response:** We agree with the reviewer that this is an important piece of social media strategy that also deserves discussion. Therefore, we have added commentary on this in Tip 3 of the revised manuscript.

**Reviewer comment 3:** Consider using "hashtags" on**** **tip 5** (Bhayani RK; et al., 2020 [Ref 1])

**Response:** We now have included hashtags in our list of strategic elements to boost audience engagement within Tip 5 of the revised manuscript. We also include this recommendation within the text of Tip 7. We thank the reviewer for this suggestion.

**Reviewer comment 4:** Re: **tips 2 and 8**- consider a recommendation for implicit bias training for all members of the team, consider a 'code of conduct' for members to sign 

**Response:** We share the reviewer’s thoughts regarding the importance of bias training and formalized conduct documents as part of a successful and inclusive SoMe strategy. We have amended tip 8 to include this recommendation in the revised document and thank the reviewer for this important addition.

**Reviewer comment 5:** - Re: **tip 7**-- "Lists can be used to facilitate intentional interaction"- it isn't clear to me what this means-- what type of lists??

**Response:** Thank you for pointing this out. We have updated this sentence to enhance the clarity of our point with this sentence, which reflects a systematic way to ensure interaction with other relevant accounts on SoMe platforms.

**Reviewer comment 6:** Re: online safety for**** **Tip 9 **would consider commenting about dealing with bullies and monitoring the accounts for spam.  For this tip might also consider the importance of a disclaimer- "this isn't medical advice" or something similar.

**Response:** We thank the reviewer for these important additions to our pitfalls of SoMe section and have included them in tip 9 of the updated manuscript.

**Reviewer comment 7:** For the conclusions you reference an increased national presence and recognition attributed to the SoMe strategy- do you have the data / graphs to support this?

**Response:** We thank the reviewer for this question. This is very difficult to quantify given multiple factors beyond our control. We have clarified this in the conclusion with the following revision: *“Because individual members of a division also influence their visibility through their academic contributions and professional activities, it is impossible to know to what degree a divisional strategy plays in individual successes.  Anecdotally, in the first five years of the division’s SoMe strategy, UNMC ID saw an increase in national speakers and national society committee members among faculty (particularly in early-career faculty), several new faculty and fellow candidates recruited through SoMe, and the publication of multiple SoMe scholarly works in the literature* *^15,29,35^* *.  The SoMe strategy may have potentially contributed to achieving the goal of increased divisional national presence and recognition by highlighting individual faculty accomplishments and raising the visibility of their hard-earned talent and expertise*.”
